# Immunomodulatory Effects of 1,25-Dihydroxyvitamin D_3_ on Dendritic Cells Promote Induction of T Cell Hyporesponsiveness to Myelin-Derived Antigens

**DOI:** 10.1155/2016/5392623

**Published:** 2016-09-14

**Authors:** Wai-Ping Lee, Barbara Willekens, Patrick Cras, Herman Goossens, Eva Martínez-Cáceres, Zwi N. Berneman, Nathalie Cools

**Affiliations:** ^1^Laboratory of Experimental Hematology, Vaccine & Infectious Disease Institute (VAXINFECTIO), Faculty of Medicine and Health Sciences, University of Antwerp, 2610 Antwerp, Belgium; ^2^Department of Neurology, Antwerp University Hospital, 2650 Edegem, Belgium; ^3^Department of Neurology, Born Bunge Institute, Translational Neurosciences, Faculty of Medicine and Health Sciences, University of Antwerp, 2610 Antwerp, Belgium; ^4^Laboratory of Medical Microbiology, Vaccine & Infectious Disease Institute (VAXINFECTIO), Faculty of Medicine and Health Sciences, University of Antwerp, 2610 Antwerp, Belgium; ^5^Division of Immunology, Germans Trias i Pujol University Hospital and Research Institute, Campus Can Ruti and Department of Cell Biology, Physiology and Immunology, The Autonomous University of Barcelona, 08913 Bellaterra, Spain; ^6^Center for Cell Therapy and Regenerative Medicine, Antwerp University Hospital, 2650 Edegem, Belgium

## Abstract

While emerging evidence indicates that dendritic cells (DC) play a central role in the pathogenesis of multiple sclerosis (MS), their modulation with immunoregulatory agents provides prospect as disease-modifying therapy. Our observations reveal that 1,25-dihydroxyvitamin D_3_ (1,25(OH)_2_D_3_) treatment of monocyte-derived DC results in a semimature phenotype and anti-inflammatory cytokine profile as compared to conventional DC, in both healthy controls and MS patients. Importantly, 1,25(OH)_2_D_3_-treated DC induce T cell hyporesponsiveness, as demonstrated in an allogeneic mixed leukocyte reaction. Next, following a freeze-thaw cycle, 1,25(OH)_2_D_3_-treated immature DC could be recovered with a 78% yield and 75% viability. Cryopreservation did not affect the expression of membrane markers by 1,25(OH)_2_D_3_-treated DC nor their capacity to induce T cell hyporesponsiveness. In addition, the T cell hyporesponsiveness induced by 1,25(OH)_2_D_3_-treated DC is antigen-specific and robust since T cells retain their capacity to respond to an unrelated antigen and do not reactivate upon rechallenge with fully mature conventional DC, respectively. These observations underline the clinical potential of tolerogenic DC (tolDC) to correct the immunological imbalance in MS. Furthermore, the feasibility to cryopreserve highly potent tolDC will, ultimately, contribute to the large-scale production and the widely applicable use of tolDC.

## 1. Introduction

Multiple sclerosis (MS) is a chronic inflammatory and neurodegenerative disease of the central nervous system (CNS) characterized by disseminated patches of demyelination and axonal loss in the brain and spinal cord. Although both genetic [1] and environmental [2] factors have been demonstrated to contribute to the onset of disease, it is currently generally accepted that MS is a T helper type 1 (Th1) and Th17-driven immune-mediated disease. This was demonstrated by immune cell infiltration and accompanying inflammatory processes leading to damage of myelin [3, 4]. Moreover, Th1 and Th17 lineage-specific cytokines, interferon-*γ* (IFN-*γ*), and interleukin-17 (IL-17) play a pivotal role in the pathogenesis of MS. Production of IFN-*γ* and IL-17 by T cells has been associated with disease activity in MS patients [4] and these cytokines are also expressed in brain lesions [5, 6]. Several clinical trials have been performed to determine if targeting effector T cells may be beneficial for MS patients. In particular, anti-IFN-*γ* therapy showed promising results in a small clinical trial in MS [7] but was not beneficial in experimental autoimmune encephalomyelitis (EAE), an animal model of MS. Hence, since IFN-*γ* and IL-17 are probably not the critical determinants of whether an effector T cell is capable of trafficking to the CNS and inducing inflammatory demyelination, the focus of research on effector T cells in MS should be on upstream pathways driving Th1 and Th17 cells. In this perspective, dendritic cells (DC), professional antigen-presenting cells, play an important role in polarizing the T cell response, thereby regulating the balance between immunity and tolerance. The possibility of modulating the function of DC using various biological or pharmacological agents makes DC interesting not only from an immunopathogenic point of view but also from a therapeutic perspective [8].

The identification of so-called tolerogenic, that is, tolerance-inducing, DC (tolDC) has paved the way for novel forms of cell-based tolerance-inducing therapies (CTT). TolDC can be characterized by low expression levels of costimulatory molecules, low production of proinflammatory cytokines, high secretion of anti-inflammatory cytokines, and a maturation-resistant phenotype [9, 10]. Importantly, tolDC can inhibit or suppress T cell responses via a multitude of mechanisms, including T cell deletion, T cell anergy, cytokine deviation, and/or the induction of regulatory T cells (Treg) [11]. In doing so, tolDC can reprogramme or modulate the immune system in order to reestablish self-tolerance in autoimmunity.

Various immunomodulatory strategies have been used to generate tolDC* in vitro*. In this respect, an exponentially increasing amount of studies is currently investigating the capacity of 1,25-dihydroxyvitamin D_3_ (1,25(OH)_2_D_3_), the active form of vitamin D_3_ [12–15]. Increasing evidence has highlighted the potential immunoregulatory functions of 1,25(OH)_2_D_3_ including the capability of 1,25(OH)_2_D_3_ to modulate both innate and adaptive immune responses [16, 17]. In particular, it was demonstrated that treatment of DC with 1,25(OH)_2_D_3_ renders DC in a semimature state, as evidenced by low expression levels of costimulatory molecules, such as CD40, CD80, and CD86, increased IL-10 production, and impaired IL-12 secretion. Consequently, 1,25(OH)_2_D_3_-treated DC display a reduced capacity to activate T cells [12–14, 18] and promising results were obtained following their administration in preclinical models of autoimmunity [15, 19–21].

So far, the first clinical trials evaluating the use of tolDC have been recently completed for type 1 diabetes, rheumatoid arthritis, and Crohn's disease [22–25]. The results were promising and the use of tolDC was safe and well tolerated. Nevertheless, several challenges still remain. First, it can be envisaged that, following migration to the inflamed tissues* in vivo*, clinically administered tolDC may acquire an immunostimulatory state upon encounter of inflammatory mediators. Hence, a stable maturation-resistant phenotype of tolDC should be aimed for. Similarly, tolDC-mediated T cell hyporesponsiveness should be persistent and robust following* in vivo* rechallenge with proinflammatory stimuli. Moreover, T cell hyporesponsiveness should be directed to disease-specific antigens, while preserving T cell capacity to respond to unrelated antigens. Other remaining issues are dose, timing, route, and frequency of administration of tolDC. Regarding the latter, it was recently demonstrated that although murine tolDC were able to reduce disease activity in EAE, the clinical effect was transient but could be restored following a subsequent injection with tolDC [26] suggesting that repeated administration is necessary. For this, large numbers of DC manufactured in accordance with current good manufacturing practice (cGMP) guidelines are required. Since the manufacturing of a large number of DC is time-consuming and cost-intensive, cryopreservation of tolDC in ready-to-use aliquots for clinical application would significantly improve the feasibility of consecutive injections. Moreover, production of sufficient numbers of DC at one time point would not only facilitate the use of DC in clinical trials but also reduce batch-to-batch variations. Whereas an efficient cryopreservation method for tolDC would greatly contribute to their use in clinical trials, studies demonstrating the influence of cryopreservation on the properties of tolDC are scarce.

In the present study, the effects of the active form of vitamin D_3_ on the differentiation, maturation, and function of monocyte-derived DC (mo-DC) from healthy controls as well as from MS patients were investigated. Given the risk of concomitant DC activation in a proinflammatory microenvironment* in vivo*, the* in vitro* stability of the maturation-resistant phenotype was also analyzed. Finally and importantly, we addressed the feasibility to cryopreserve tolDC by assessing the effects of cryopreservation on the phenotype and allogeneic T cell-stimulatory capacity of 1,25(OH)_2_D_3_-treated DC.

## 2. Material and Methods

### 2.1. Study Population

Peripheral blood from healthy volunteers was obtained from buffy coats provided by the Antwerp Blood Transfusion Center (Red Cross-Flanders, Edegem, Belgium). MS patients, diagnosed according to the revised McDonald criteria [27], were recruited by the Department of Neurology from the Antwerp University Hospital (Edegem, Belgium). Ten patients (6 males and 4 females) with an average age of 38 years (range: 25–52 years) and a median expanded disability status scale (EDSS) score of 3 (range: 0–5) were included (Table 1). All subjects gave written consent after they were informed of the nature and possible risks of the study. The study was approved by the Ethics Committee of the Antwerp University Hospital and followed the tenets of the Declaration of Helsinki. Approximately 100 mL of heparinized blood was collected by venous puncture. Samples were processed within 24 hours after collection.

### 2.2. Dendritic Cell Culture

**Figure 1:**
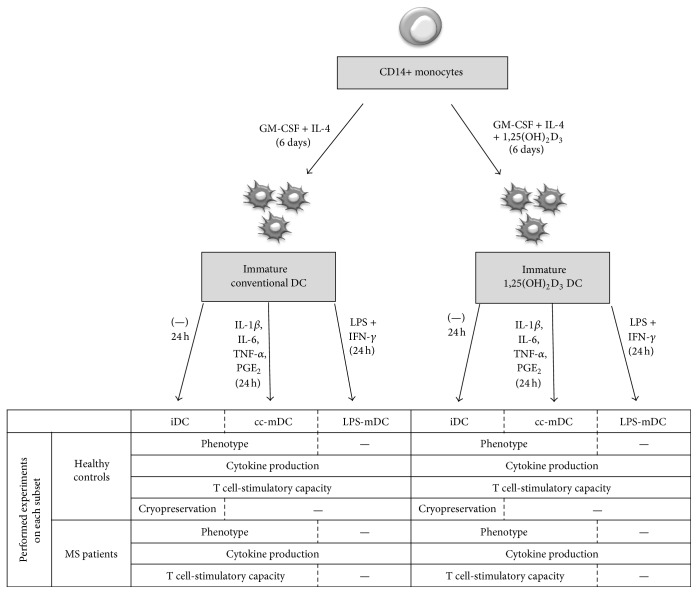
Experimental design. CD14+ monocytes were cultured for 6 days in the presence of IL-4 and GM-CSF or in the presence of IL-4, GM-CSF, and 1,25(OH)_2_D_3_ to obtain immature conventional DC (iDC) or 1,25(OH)_2_D_3_-treated iDC, respectively. On day 6, iDC were stimulated with a cocktail of proinflammatory cytokines (i.e., cc-matured DC (cc-mDC)) or with LPS and IFN-*γ* (i.e., LPS-matured DC (LPS-mDC)) or left untreated (i.e., iDC).

Peripheral blood mononuclear cells (PBMC) were isolated by density gradient centrifugation (Ficoll-Paque*™* PLUS, GE Healthcare, Chalfont St. Giles, UK). Next, CD14+ monocytes were purified by CD14+ immunomagnetic selection (CD14 Reagent, Miltenyi Biotec, Bergisch Gladbach, Germany), according to manufacturer's instructions, and were directly used for* in vitro* DC differentiation (Figure 1). The CD14-depleted cell fraction (i.e., peripheral blood lymphocytes (PBL)) was cryopreserved in freezing solution containing 90% fetal bovine serum (Life Technologies, Paisley, UK) supplemented with 10% dimethyl sulfoxide (DMSO, Sigma-Aldrich, Bornem, Belgium) and stored at −80°C for later use in DC/T cell cocultures. In order to generate immature conventional DC, CD14+ monocytes were cultured at a density of 1–1.2 × 10^6^/mL for 7 days in Iscove's modified Dulbecco's Medium (IMDM with L-glutamine, Life Technologies) supplemented with 10 *µ*g/mL gentamicin (Life Technologies), 1 *µ*g/mL amphotericin B (Life Technologies), 2.5% heat-inactivated human (h) AB serum (Life Technologies), 25 ng/mL IL-4 (Gentaur, Brussels, Belgium), and 17.5 ng/mL granulocyte macrophage colony-stimulating factor (GM-CSF, Gentaur). Simultaneously, tolDC were differentiated under the same conditions, but with the addition of 10^−5^ M 1,25(OH)_2_D_3_ (Sigma-Aldrich). Cells were replenished on day 3 with fresh medium and cytokines. On day 6, DC were (i) stimulated for 24 hours by adding a cocktail of proinflammatory cytokines consisting of 100 U/mL IL-1*β* (Biosource Europe, Nivelles, Belgium), 500 U/mL IL-6 (Life Technologies), 2.5 ng/mL tumor necrosis factor-*α* (TNF-*α*) (Gentaur), and 10^−7^ M prostaglandin E_2_ (PGE_2_, Prostin E_2_®, Pfizer, Elsene, Belgium) (i.e., cytokine cocktail-matured DC (cc-mDC)), or (ii) stimulated for 24 hours by adding 1 *µ*g/mL lipopolysaccharide (LPS) (Invivogen, San Diego, CA, USA) and 1000 IU/mL IFN-*γ* (ImmunoTools, Friesoythe, Germany) (i.e., LPS-matured DC (LPS-mDC)), or (iii) left untreated (i.e., immature DC (iDC)). Cells were cultured in a humidified atmosphere with 5% CO_2_ at 37°C. On day 7, conventional and 1,25(OH)_2_D_3_-treated DC were harvested and used in further experiments.

### 2.3. Cryopreservation and Thawing Conditions

On day 7, immature conventional and 1,25(OH)_2_D_3_-treated DC were resuspended in freezing medium containing 86% hAB serum, 10% DMSO, and 4% glucose and frozen in 2 mL cryotubes (Sarstedt, Numbrecht, Germany) at a concentration of 10^7^ cells/mL. Cell suspensions were slowly frozen at a cooling rate of −1°C/min to −80°C by using a Mr. Frosty freezing container (Nalgene, Rochester, USA). Within 4 days, cell suspensions were transferred to liquid nitrogen for long-term storage. Frozen samples were quickly thawed at 37°C in a warm water bath and subsequently transferred into preheated (37°C) CellGro medium (CellGenix, Freiburg, Germany) supplemented with 1% hAB serum. Next, cells were washed and resuspended in preheated CellGro medium supplemented with 1% hAB serum, 25 ng/mL IL-4, and 17.5 ng/mL GM-CSF. Following a 2 h resting phase at 37°C in an ultralow adherent 6-well plate, conventional and 1,25(OH)_2_D_3_-treated iDC were stimulated with a proinflammatory cytokine cocktail or left untreated. After 24 hours, cells were harvested and used in further experiments.

### 2.4. Flow Cytometric Immunophenotyping

For phenotypic characterization of DC, direct immunofluorescence staining was performed using the following fluorochrome-labeled mouse anti-human monoclonal antibodies: anti-CD86-fluorescein isothiocyanate (FITC) (BD Pharmingen, Erembodegem, Belgium), anti-CD80-phycoerythrin (PE) (BD Biosciences, Erembodegem, Belgium), anti-human leukocyte antigen- (HLA-) DR-peridinin chlorophyll (PerCP) (BD Biosciences), anti-CD83-FITC (Life Technologies), anti-dendritic cell-specific intercellular adhesion molecule-3-grabbing nonintegrin- (DC-SIGN-) PE (BD Pharmingen), anti-CD14-PerCP (BD Biosciences), anti-programmed death-ligand 1- (PD-L1-) FITC (BD Pharmingen), anti-CCR7-PE (R&D Systems, Abingdon, UK), and anti-immunoglobulin-like transcript 3- (ILT3-) PE-Cy5 (Immunotech, Marseille, France). Isotype-matched control monoclonal antibodies were used to determine nonspecific background staining. Propidium iodide staining was done for analysis of cell viability. For analytical flow cytometry, at least 10^4^ events were analyzed using a BD FACScan flow cytometer (BD Biosciences). All results were analyzed using FlowJo software (Tree Star, Ashland, USA).

### 2.5. Cytokine Release Assays

For quantitative detection of the cytokine secretion profile of the different DC populations, a multiplex fluorescent bead immunoassay (IL-1*β*, IL-2, IL-4, IL-5, IL-6, IL-8, IL-10, IL-12p70, TNF-*α*, TNF-*β*, and IFN-*γ*) (Bender MedSystems, Vienna, Austria) and a transforming growth factor-*β* (TGF-*β*) ELISA (eBioscience, San Diego, United States of America) were used according to the manufacturer's instructions. For this, iDC and mDC were harvested, washed, and resuspended in IMDM supplemented with 5% hAB serum at a concentration of 5 × 10^5^ cells/mL. After 24 hours, supernatant was collected for analysis of cytokine production.

### 2.6. Allogeneic Mixed Lymphocyte Reaction (Allo-MLR)

In order to assess the allogeneic T cell-stimulatory capacity of DC, DC were cocultured with allogeneic responder PBL at a 1 : 10 ratio. Nonstimulated responder PBL served as negative control, while allogeneic responder cells stimulated with mitomycin C-treated (Sigma-Aldrich) PBL were used as positive control. Cocultures were performed in IMDM supplemented with 5% hAB serum at 37°C. After 6 days, the secreted level of IFN-*γ* in the cell culture supernatant was determined as a measure for allostimulatory capacity using a commercially available ELISA kit (PeproTech, New Jersey, USA), where each condition is measured in triplicate.

### 2.7. Antigen-Specific T Cell-Stimulatory Capacity of DC

In order to determine the antigen-specific T cell-stimulatory capacity of DC, 5 × 10^6^ PBL were stimulated with a pool of myelin-derived peptides (5 *µ*g/mL myelin oligodendrocyte glycoprotein (MOG) (aa 1–22), 5 *µ*g/mL MOG (aa 34–56), 5 *µ*g/mL MOG (aa 64–86), and 5 *µ*g/mL MOG (aa 74–96) and 5 *µ*g/mL myelin basic protein (MBP) (aa 84–102) and 5 *µ*g/mL MBP (aa 143–168), all purchased from Severn Biotech Ltd. (Kidderminster, UK)) in the presence or absence of 5 × 10^5^ autologous DC. After 7 days of coculture, PBL were analyzed for antigen-specific responsiveness by determining IFN-*γ* production following antigenic restimulation by means of IFN-*γ* ELISPOT (Mabtech, Nacka Strand, Sweden), according to the manufacturer's instructions. In brief, 2 × 10^5^ stimulated PBL were rechallenged with 5 *µ*g/mL of MOG- and MBP-derived peptides in anti-IFN-*γ* antibody-coated 96-well polyvinylidene fluoride (PVDF) plates (Millipore, Bedford, MA, USA). Nonstimulated PBL were used as a control and each condition was measured in quadruple. In some experiments, PBL were harvested on day 7 of coculture and restimulated either with 0.5 *µ*g/mL cytomegalovirus (CMV) pp65-derived peptide pool or with 5 *µ*g/mL of MOG- and MBP-derived peptides combined with cryopreserved fully mature conventional DC of the same donor. Frequencies of antigen-specific IFN-*γ*-secreting cells were calculated based on the number of spots counted using an automated AID ELISPOT Reader system (AID GmbH, Strassberg, Germany) and analyzed using AID ELISPOT software version 5.0. A positive responder was defined according to the guidelines of the ELISPOT proficiency panel from the Cancer Vaccine Consortium [28]: per 10^6^ PBL, the mean antigen-specific spot count for a donor and condition must be greater than or equal to 15 spots per well and at least 2.5 times as high as the background reactivity.

### 2.8. DC-Mediated Induction of Suppressive T Cell Populations

The induction of different populations of Treg was determined following coculture of autologous PBL, stimulated with MOG- and MBP-derived peptides in the presence or absence of DC, as described above. At day 6, 10 *µ*g/mL brefeldin A (GolgiStop, BD Pharmingen) was added to the DC/T cell coculture and incubated overnight at 37°C. Next, cells were harvested and membrane markers were stained with the following mouse anti-human monoclonal antibodies: anti-CD3-PerCP-Cy5.5 (BD Biosciences), anti-CD4-allophycocyanin-H7 (anti-CD4-APC-H7) (BD Biosciences), anti-CD8-Pacific Blue (Life Technologies), and anti-CD25-PE-Cy7 (BD Biosciences). Subsequently, cells were fixed and permeabilized using a FOXP3 Staining Buffer Kit (eBioscience, Hatfield, UK), according to manufacturer's instructions, and intracellular markers were stained with anti-FOXP3-alexa488 (BD Pharmingen), anti-TGF-*β*-PE (IQ Products, Groningen, Netherlands), and anti-IL-10-APC (BD Pharmingen). Labeled cells were analyzed on a Cyflow ML flow cytometer (Partec, Münster, Germany). For analytical flow cytometry, at least 5 × 10^4^ CD3+ CD4+ CD8− lymphocytes were acquired. All results were analyzed using FlowJo software.

### 2.9. Statistical Analysis

Results are expressed as mean ± standard error of mean (SEM), unless stated otherwise. Comparisons were validated using one-way or two-way analysis of variance (ANOVA) with a Bonferroni* post hoc* test for pairwise group comparisons, when appropriate using GraphPad version 5 software (Prism, La Jolla, CA, USA). A* p *value of ≤0.05 was considered as statistically significant.

## 3. Results

### 3.1. 1,25(OH)_2_D_3_-Treated Immature DC Express Lower Levels of CD86 and HLA-DR and Display an Anti-Inflammatory Cytokine Profile as Compared to Conventional DC

**Figure 2:**
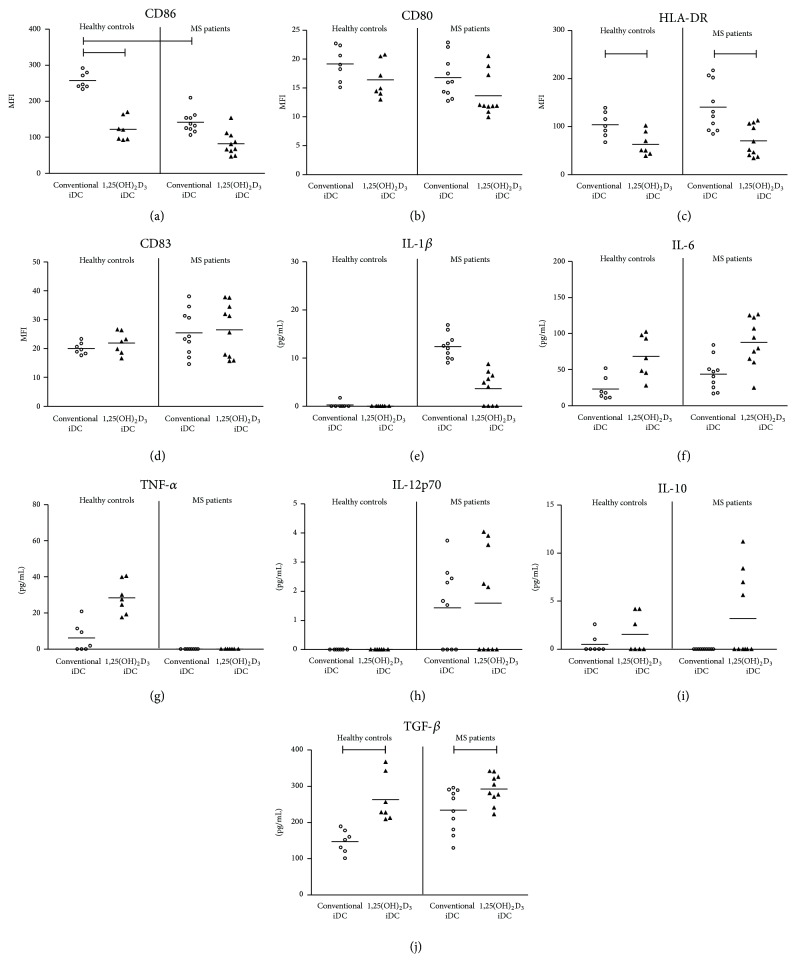
Characterization of* in vitro* differentiated iDC from healthy controls and MS patients. CD14+ monocytes were cultured for 7 days in the presence of IL-4 and GM-CSF or in the presence of IL-4, GM-CSF, and 1,25(OH)_2_D_3_ to obtain conventional iDC (open dots) or 1,25(OH)_2_D_3_-treated iDC (filled triangles), respectively. The expression of (a) CD86, (b) CD80, (c) HLA-DR, and (d) CD83 by DC of healthy controls (*n* = 7) and MS patients (*n* = 10) is determined by flow cytometry. Cytokine secretion of (e) IL-1*β*, (f) IL-6, (g) TNF-*α*, (h) IL-12p70, (i) IL-10, and (j) TGF-*β* by conventional iDC and 1,25(OH)_2_D_3_-treated iDC is determined by a multiplex immunoassay or ELISA. Horizontal lines show the mean. MFI, mean fluorescence intensity, and iDC, immature DC.

Previously, we reported no major differences in the phenotype of* in vitro* generated immature DC of MS patients as compared to those of healthy controls, except for the expression of the migration marker CCR7 [29]. Here, we demonstrate that 1,25(OH)_2_D_3_ treatment of immature mo-DC from healthy controls results in significantly lower expression levels of CD86 and of HLA-DR as compared to conventional DC, while 1,25(OH)_2_D_3_-treated mo-DC from MS patients only show lower expression levels of HLA-DR as compared to conventional mo-DC (Figures 2(a) and 2(c)). However, it needs to be noted that immature conventional DC of healthy controls show a significantly higher expression level of CD86 as compared to those of MS patients. MS-derived mo-DC show lower expression levels of DC-SIGN following 1,25(OH)_2_D_3_ treatment, despite the fact that both conventional and 1,25(OH)_2_D_3_-treated mo-DC from MS patients display significantly higher expression levels of DC-SIGN as compared to those of healthy controls (Supplementary Figure  1, in Supplementary Material available online at http://dx.doi.org/10.1155/2016/5392623). No differences for the expression of CD80 and CD83 were observed following 1,25(OH)_2_D_3_ treatment of mo-DC from both healthy controls and MS patients (Figures 2(b) and 2(d)). Furthermore, 1,25(OH)_2_D_3_ treatment did not affect the expression of CD14, of the chemokine receptor CCR7, and of the inhibitory molecules PD-L1 and ILT-3 by mo-DC from both healthy controls and MS patients (Supplementary Figure  1).

Subsequently, the cytokine secretion profile of both conventional and 1,25(OH)_2_D_3_-treated immature mo-DC from healthy controls and MS patients was assessed using a multiplex immunoassay and ELISA. No major differences regarding the cytokine secretion profile of mo-DC from MS patients as compared to mo-DC from healthy controls could be detected (Figures 2(e)–2(j)). Remarkably, immature DC from healthy controls as well as from MS patients produced more TGF-*β* following 1,25(OH)_2_D_3_ treatment as compared to conventional DC (Figure 2(j)).

### 3.2. 1,25(OH)_2_D_3_-Treated DC Display a Semimature Phenotype

**Figure 3:**
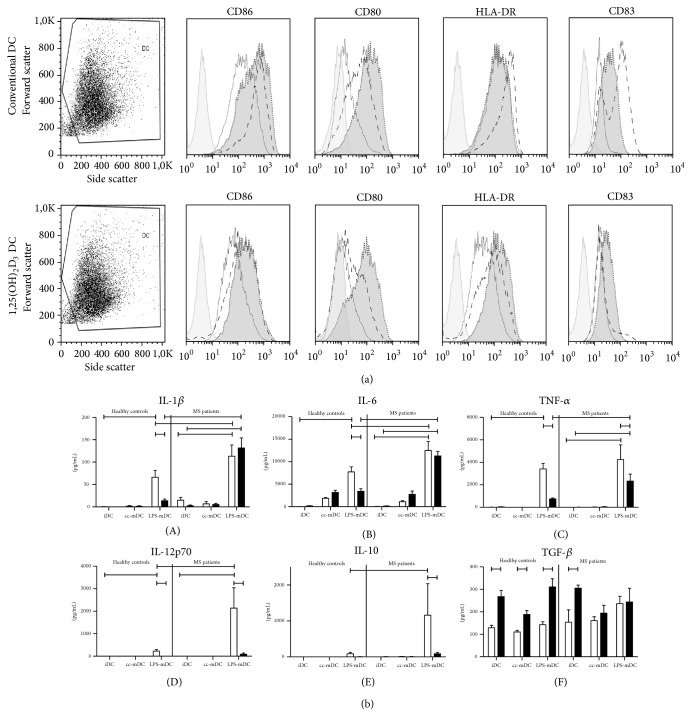
Immunophenotypic analysis and cytokine secretion profile of* in vitro* differentiated DC from healthy controls and MS patients upon maturation with proinflammatory stimuli. CD14+ monocytes were cultured for 6 days in the presence of IL-4 and GM-CSF or in the presence of IL-4, GM-CSF, and 1,25(OH)_2_D_3_ to obtain immature conventional DC (iDC) or 1,25(OH)_2_D_3_-treated iDC, respectively. On day 6, DC were stimulated with a cocktail of proinflammatory cytokines (i.e., cc-mDC) or with LPS and IFN-*γ* (i.e., LPS-mDC) or left untreated (i.e., iDC). (a) Representative example showing immunophenotypic analysis of DC. The expression of CD86, CD80, HLA-DR, and CD83 by conventional DC and 1,25(OH)_2_D_3_-treated DC is determined by flow cytometry. Immature DC are represented by a solid line, cc-mDC are represented by a dashed line, and LPS-mDC are represented by a dark grey filled histogram. Isotype-matched controls are represented by the light grey filled histograms. For analysis, DC were gated on light scatter properties as depicted in the forward scatter (FSC) versus side scatter (SSC) dot plot. (b) Cytokine secretion of (A) IL-1*β*, (B) IL-6, (C) TNF-*α*, (D) IL-12p70, (E) IL-10, and (F) TGF-*β* by conventional DC (open bars) and 1,25(OH)_2_D_3_-treated DC (black bars) is determined by a multiplex immunoassay or ELISA. Results are expressed as mean ± SEM (healthy controls: *n* = 7; MS patients: *n* = 10).

Next, immature mo-DC were stimulated with a cocktail of proinflammatory cytokines (i.e., cc-mDC) for 24 hours. Conventional DC of both healthy controls (Table 2(a)) and MS patients (Table 2(b)) acquire a mature phenotype following activation with proinflammatory stimuli, as evidenced by upregulation of the expression of CD80, CD86, CD83, and HLA-DR (Figure 3(a)). Importantly, 1,25(OH)_2_D_3_-treated DC from both healthy controls and MS patients displayed a significantly lower expression of CD86, CD83, and HLA-DR upon stimulation with a proinflammatory cytokine cocktail in comparison with conventional DC. However, also 1,25(OH)_2_D_3_-treated DC underwent a maturation process as demonstrated by upregulated expression of CD80, CD83, CD86, and HLA-DR, albeit less pronounced as in conventional DC. No significant differences could be observed regarding the expression of DC-SIGN, CD14, and the inhibitory molecules ILT-3 and PD-L1 between 1,25(OH)_2_D_3_-treated mo-DC and conventional mo-DC following stimulation with proinflammatory molecules (Supplementary Figure  1).

Additionally, we investigated the cytokine secretion profile of conventional and 1,25(OH)_2_D_3_-treated mo-DC following stimulation with proinflammatory molecules. Our findings indicate significantly higher levels of secreted IL-1*β*, IL-6, IL-12p70, and TNF-*α* by conventional DC of both healthy controls and MS patients following stimulation with LPS and IFN-*γ* (Figure 3(b)). Noteworthy, LPS and IFN-*γ*-stimulated conventional mo-DC from MS patients secrete significantly higher, in particular 20-fold more, amounts of IL-12p70 as compared to conventional mo-DC from healthy controls. Similarly, also the secretion of IL-1*β* and IL-6 by mo-DC from MS patients was increased as compared to mo-DC from healthy controls. Importantly, 1,25(OH)_2_D_3_ treatment of mo-DC drastically abrogated the secretion of IL-12p70 and TNF-*α* by mo-DC from both healthy controls and MS patients. Production of IL-1*β* and IL-6 following stimulation with LPS and IFN-*γ* was only reduced in 1,25(OH)_2_D_3_-treated mo-DC from healthy controls as compared to conventional mo-DC. Even following 1,25(OH)_2_D_3_ treatment, mo-DC from MS patients display a significantly higher secretion of IL-1*β*, IL-6, and TNF-*α* following stimulation with LPS and IFN-*γ* as compared to 1,25(OH)_2_D_3_-treated mo-DC of healthy controls. In our hands, we could not observe IL-10 secretion by 1,25(OH)_2_D_3_-treated mo-DC from either healthy controls or MS patients. However, following stimulation with LPS and IFN-*γ*, the secretion of IL-10 by conventional mo-DC of MS patients was significantly higher as compared to 1,25(OH)_2_D_3_-treated mo-DC.

In summary, our findings demonstrate that 1,25(OH)_2_D_3_ treatment of mo-DC renders DC of both healthy controls and MS patients in a semimature state as indicated by a significantly impaired upregulation of the expression of costimulatory molecules and activation markers as well as by a significantly reduced secretion of proinflammatory cytokines.

### 3.3. Cryopreservation Did Not Affect the Expression of Membrane Markers by 1,25(OH)_2_D_3_-Treated DC

**Figure 4:**
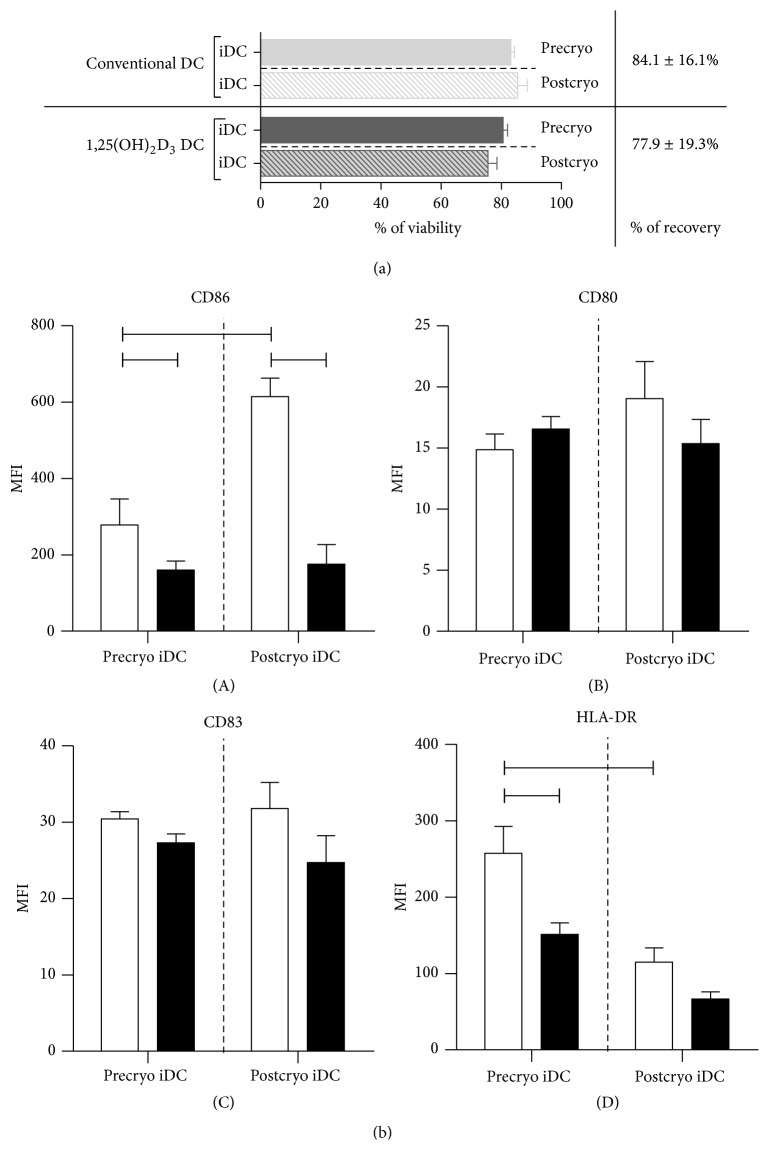
Viability, recovery, and phenotypic characteristics of conventional and 1,25(OH)_2_D_3_-treated iDC from healthy controls before and after cryopreservation. CD14+ monocytes were cultured for 7 days in the presence of IL-4 and GM-CSF or in the presence of IL-4, GM-CSF, and 1,25(OH)_2_D_3_ to obtain immature conventional DC or 1,25(OH)_2_D_3_-treated DC, respectively. On day 7, iDC were frozen (i.e., precryo iDC). Following a 2 h resting phase after thawing, iDC were left untreated for 24 h (i.e., postcryo iDC). (a) Viability of conventional and 1,25(OH)_2_D_3_-treated iDC of healthy controls (*n* = 5) was determined on day 7 of DC culture (i.e., precryo) and 26 h after thawing (i.e., postcryo). Recovery is expressed as the ratio of cells harvested before and after cryopreservation. (b) The MFI of (A) CD86, (B) CD80, (C) CD83, and (D) HLA-DR by immature conventional DC (open bars) or 1,25(OH)_2_D_3_-treated DC (black bars) of healthy controls (*n* = 5) was determined. Results are expressed as mean ± SEM. MFI, mean fluorescence intensity, and iDC, immature DC.

In order to facilitate multiple injections with tolDC for clinical applications, we evaluated the feasibility to cryopreserve tolDC. For this, viability, recovery, and phenotype of cryopreserved iDC were assessed upon thawing. We demonstrate a yield of 78% and a viability of 75% of immature 1,25(OH)_2_D_3_-treated DC following a freeze-thaw cycle (Figure 4(a)). No significant differences for the yield and viability were found between conventional DC and 1,25(OH)_2_D_3_-treated DC. Furthermore, while conventional iDC display a significantly increased expression of CD86 and decreased expression of HLA-DR following cryopreservation, underscoring the plasticity of the phenotypic characteristics of conventional DC, no differences regarding the expression levels of HLA-DR, CD80, CD86, and CD83 were observed for 1,25(OH)_2_D_3_-treated mo-DC following cryopreservation (Figure 4(b)).

In order to determine the ability of 1,25(OH)_2_D_3_-treated iDC to maintain their semimature phenotype after cryopreservation, iDC were stimulated with a proinflammatory cytokine cocktail for 24 hours following a 2 h resting phase after thawing. Conventional DC display upregulated expression of CD83 (Table 3). No significant differences were detected for the expression of HLA-DR, CD80, and CD86, despite the fact that marked upregulation of CD86 expression by conventional DC was already observed following cryopreservation (Figure 4(b)). In addition, cryopreservation did not affect the expression profile of membrane markers by 1,25(OH)_2_D_3_-treated mo-DC, not even upon stimulation with proinflammatory signals, indicative of a robust semimature phenotype of 1,25(OH)_2_D_3_-treated DC (Table 3).

### 3.4. Allogeneic T Cell-Stimulatory Capacity of 1,25(OH)_2_D_3_-Treated DC before and after Cryopreservation

**Figure 5:**
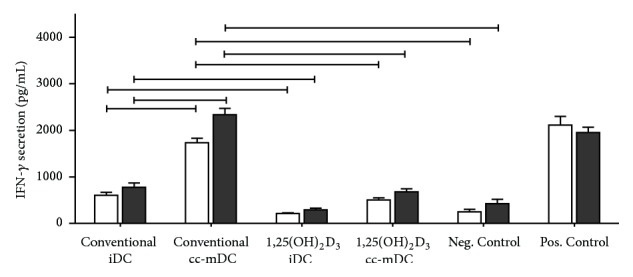
Allogeneic T cell-stimulatory capacity of 1,25(OH)_2_D_3_-treated DC before and after cryopreservation. Fresh and frozen iDC and mDC were cocultured with allogeneic responder PBL at a 1 : 10 ratio. Nonstimulated PBL served as negative control, while allogeneic responder cells stimulated by mitomycin C-treated PBL (10 : 1 responder/stimulator ratio) were used as positive control. After 6 days, cell culture supernatant was collected and the secreted level of IFN-*γ* was used as a measure for allostimulatory capacity by means of IFN-*γ* ELISA. Each condition was measured in triplicate. Results of healthy controls (*n* = 5) are expressed as mean ± SEM. The open bars and black bars represent the measured IFN-*γ* secretion before cryopreservation and after cryopreservation, respectively. iDC, immature DC; cc-mDC, cytokine cocktail-matured DC; and PBL, peripheral blood lymphocytes.

The immunostimulatory capacity of conventional and 1,25(OH)_2_D_3_-treated DC was determined in an allogeneic mixed leukocyte reaction (allo-MLR). For this, responder PBL were stimulated with allogeneic iDC or mDC of healthy controls at a 10 : 1 ratio. The level of IFN-*γ* secreted in the coculture supernatant was used as a measure for allogeneic T cell-stimulatory capacity. As demonstrated in Figure 5, conventional mo-DC have profound capacity to stimulate IFN-*γ*-production by responder PBL in an allo-MLR, which is not affected by cryopreservation of mo-DC, as compared to the negative control. In contrast, no allogeneic IFN-*γ* production is induced by responder PBL following stimulation with 1,25(OH)_2_D_3_-treated DC, irrespective of the maturation state of DC. Importantly, this T cell hyporesponsiveness was retained following stimulation with cryopreserved allogeneic 1,25(OH)_2_D_3_-treated iDC or mDC.

### 3.5. 1,25(OH)_2_D_3_-Treated DC Induce Antigen-Specific T Cell Hyporesponsiveness to Myelin-Derived Antigens

**Figure 6:**
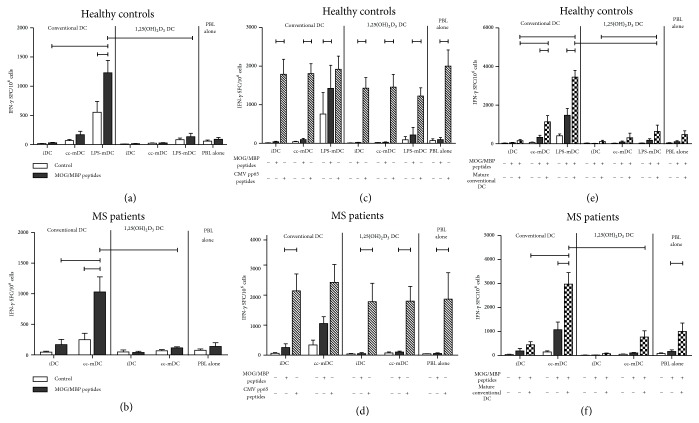
1,25(OH)_2_D_3_-Treated DC induce stable antigen-specific T cell hyporesponsiveness to myelin-derived antigens (MOG/MBP peptides) in both healthy controls and MS patients. PBL stimulated with MOG/MBP peptides with or without autologous iDC or mDC were restimulated with MOG/MBP peptides (black bars) after 7 days of initial coculture. Controls represent nonrestimulated PBL (open bars). The secretion of IFN-*γ* was used as a measure for autologous T cell-stimulatory capacity. Each condition was measured in quadruple. Results are expressed as mean ± SEM. T cell hyporesponsiveness induced by 1,25(OH)_2_D_3_-treated DC of healthy controls (*n* = 7) and MS patients (*n* = 4) is shown, respectively, in (a) and (b). The antigen specificity of T cell hyporesponsiveness induced by 1,25(OH)_2_D_3_-treated DC was determined for healthy controls (c) and MS patients (d). PBL stimulated with MOG/MBP peptides with or without autologous DC were restimulated with either MOG/MBP peptides (black bars) or CMV pp65 peptides (dashed bars) after 7 days of initial coculture. ((e) and (f)) Stability of T cell hyporesponsiveness induced by 1,25(OH)_2_D_3_-treated DC of healthy individuals (e) and MS patients (f). PBL stimulated with MOG/MBP peptides with or without autologous DC were restimulated either with MOG/MBP peptides (black bars) or with MOG/MBP peptides combined with fully mature conventional DC (blocked bars) after 7 days of initial coculture. iDC, immature DC; cc-mDC, cytokine cocktail-matured DC; LPS-mDC, lipopolysaccharide-matured DC; PBL, peripheral blood lymphocytes; MOG, myelin oligodendrocyte glycoprotein; MBP, myelin basic protein; CMV, cytomegalovirus; and SFC, spot forming cells.

In order to determine the antigen-specific T cell-stimulatory capacity of conventional and 1,25(OH)_2_D_3_-treated DC of healthy controls (*n* = 7) and MS patients (*n* = 4), PBL were stimulated with myelin-derived peptides in the presence or absence of autologous iDC or mDC at a 10 : 1 ratio for 7 days. Subsequently, the number of antigen-specific IFN-*γ*-secreting T cells was determined using IFN-*γ* ELISPOT. Following rechallenge of* in vitro* stimulated PBL with myelin-derived peptides, antigen-specific IFN-*γ* production by PBL stimulated with conventional mDC was significantly higher as compared to PBL stimulated with conventional iDC (Figures 6(a) and 6(b)). Hence, stimulation with fully mature conventional DC is mandatory to detect myelin-specific IFN-*γ*-secreting T cells in both healthy controls and MS patients. Of interest, there was no significant difference in the number of MOG/MBP responders between healthy controls and MS patients. Seven out of 16 healthy controls and 4 out of 7 MS patients displayed a positive myelin-specific response following stimulation with conventional mDC, as defined in the Material and Methods. In contrast, PBL stimulated with 1,25(OH)_2_D_3_-treated mDC fail to respond to a rechallenge with myelin-derived peptides, as evidenced by the significantly reduced number of IFN-*γ*-secreting spot-forming cells as compared to PBL stimulated with conventional mDC (Figures 6(a) and 6(b)). Based on these observations, we demonstrate that 1,25(OH)_2_D_3_-treated DC from both healthy volunteers and MS patients induce T cell hyporesponsiveness, irrespective of the maturation state of DC.

In order to investigate if the T cell hyporesponsiveness mediated by 1,25(OH)_2_D_3_-treated DC is antigen-specific, we investigated the capacity of T cells stimulated with 1,25(OH)_2_D_3_-treated DC and myelin-derived peptides to respond to an unrelated antigen, that is, cytomegalovirus (CMV) pp65-derived peptides. For this, PBL stimulated with a pool of myelin-derived peptides in the presence or absence of iDC or mDC were rechallenged with myelin-derived peptides or with a CMV pp65-derived peptide pool after 7 days of initial coculture. While a low frequency of myelin-specific IFN-*γ*-secreting spot-forming cells was detected when PBL from healthy controls (Figure 6(c)) and from MS patients (Figure 6(d)) were rechallenged with myelin-derived peptides, PBL were still able to secrete IFN-*γ* production following rechallenge with a CMV pp65-derived peptide pool in all conditions tested.

### 3.6. Mode of Action of T Cell Hyporesponsiveness Mediated by 1,25(OH)_2_D_3_-Treated DC

In order to evaluate whether the T cell hyporesponsiveness mediated by 1,25(OH)_2_D_3_-treated DC could be reversed, PBL were stimulated with myelin-derived peptides in the presence or absence of autologous conventional and 1,25(OH)_2_D_3_-treated iDC or mDC for 7 days. Next, PBL were rechallenged with myelin-derived peptides alone or with myelin-derived peptides and conventional cc-mDC. In both healthy controls (Figure 6(e)) and MS patients (Figure 6(f)), inclusion of a strong stimulus, such as fully mature conventional DC, together with antigen rechallenge is associated with a significantly higher number of antigen-specific IFN-*γ*-secreting T cells as compared to PBL rechallenged with myelin-derived peptides alone. In contrast, rechallenge of PBL tolerized to myelin-derived peptides in the presence of 1,25(OH)_2_D_3_-treated mDC using conventional cc-mDC did not affect the myelin-specific T cell response. For this, we conclude that PBL stimulated with 1,25(OH)_2_D_3_-treated DC were rendered in a robust hyporesponsive state in both healthy volunteers and MS patients, irrespective of the maturation state of DC.

Since others described that CD4+ T cells primed by iDC acquire a Treg phenotype [30, 31], we assessed the presence of Treg populations in autologous DC/T cell cocultures. Hereto, PBL were stimulated with myelin-derived peptides in the presence or absence of autologous conventional or 1,25(OH)_2_D_3_-treated iDC at a 10 : 1 ratio. After 7 days of coculture, multiparametric flow cytometry was performed to characterize the presence of CD4+ CD25+ FOXP3+ Treg as well as the expression of intracellular immunosuppressive cytokines. No differences in the number of Treg following stimulation with both conventional and 1,25(OH)_2_D_3_-treated DC could be detected. In addition, we could not observe IL-10 and/or TGF-*β*-expressing cells (Table 4).

## 4. Discussion

Current disease-modifying therapies to prevent or slow progressive disability in MS include IFN-*β*, glatiramer acetate, natalizumab, and fingolimod. Recently, a number of new treatment strategies have been approved for clinical use by the regulatory authorities including teriflunomide [32], dimethyl fumarate (BG-12) [33], and alemtuzumab [34]. All are primarily aimed at reducing the number of relapses and slowing the disease progression; however, none induces a long-lasting, drug-free remission of MS, whereas several are accompanied by severe side effects such as secondary autoimmunity and infections. Therefore, continuous efforts are aimed at the development of new therapeutic approaches that specifically target the pathologic autoinflammatory processes in MS without generalized immune suppression. In this perspective, the identification of tolDC as cellular mediators to downmodulate unwanted autoimmune responses may provide new prospects. Indeed, preclinical evidence from animal models supports the therapeutic potential of tolDC as demonstrated by prevention of transplant rejection in skin and heart graft models [35, 36] or by attenuation of pathogenic T cells and reestablishment of self-tolerance following administration of* ex vivo* generated tolDC in collagen-induced arthritis (CIA), nonobese diabetic (NOD), and EAE models [15, 19–21]. These promising outcomes resulted in a number of recently completed phase I clinical trials using tolDC in patients with type 1 diabetes [22], rheumatoid arthritis [23, 25], and Crohn's disease [24]. Treatment with autologous tolDC was well tolerated and safe without any discernible adverse events or toxicities. While these studies highlight the emergence of tolDC therapy as a new approach to treat autoimmune diseases, numerous questions still remain in view of the translation of bench findings to the bedside. Indeed, although different strategies using a variety of tolerogenic agents for the generation of tolDC* in vitro* are showing promising results, not all tolerogenic agents seem to have the ability to maintain a stable tolerogenic profile, once administrated* in vivo* [37].

Here, we demonstrated that 1,25(OH)_2_D_3_ treatment renders mo-DC in a semimature state as evidenced by impaired upregulation of the expression of CD86, CD80, CD83, and HLA-DR upon stimulation with proinflammatory molecules as compared to the expression of these markers by conventional DC. Furthermore, no phenotypic differences were found between mo-DC from healthy controls and MS patients following 1,25(OH)_2_D_3_ treatment. Similar results were previously demonstrated by others [14, 38]. Additionally, while 1,25(OH)_2_D_3_-treated immature DC secrete higher levels of TGF-*β* as well as of IL-6 and TNF-*α*, as compared to conventional DC, 1,25(OH)_2_D_3_-treated DC show impaired secretion of proinflammatory cytokines following stimulation with LPS and IFN-*γ* as compared to conventional DC, except for IL-1*β* and IL-6 secretion by mature 1,25(OH)_2_D_3_-treated mo-DC from MS patients. Whereas concomitant DC activation following administration in an inflammatory microenvironment* in vivo* can be envisaged, our findings support a semimature phenotype of 1,25(OH)_2_D_3_-treated DC from both healthy controls and MS patients, as evidenced by impaired upregulation of the expression of costimulatory markers and of the secretion of proinflammatory cytokines following rechallenge with LPS, in agreement with previous observations by others [14, 38]. Nevertheless, the levels of IL-1*β*, IL-6, and TNF-*α* secreted by mature DC of MS patients are significantly higher as compared to those from healthy controls, even following treatment with 1,25(OH)_2_D_3_. Hence, careful safety monitoring will be required when administering 1,25(OH)_2_D_3_-treated DC in a clinical setting, for example, for the induction of tolerance in MS patients.

Recently, it was demonstrated that one injection with murine tolDC in EAE resulted in a profound clinical effect [26]. However, the clinical improvements were transient underscoring the possible need for multiple injections with tolerance-inducing cell products if long-lasting regulation of the autoimmune response is aimed for. Therefore, cryopreservation of DC—allowing the generation of ready-to-use aliquots—may facilitate the clinical use of tolDC. In addition, this approach can minimize batch-to-batch variations. Hence, in order to guarantee the comparability of the cell product before and after cryopreservation, the function and phenotype of the DC must be preserved after a freeze-thaw cycle. Previously, others have described and standardized a number of approaches to generate immunogenic DC from cryopreserved monocytes or PBMC [39, 40]. However, the reported effects of cryopreservation on mo-DC differentiation, function, and allogeneic T cell-stimulatory capacity are conflicting [41–43]. In addition, frozen PBMC or monocytes require additional manipulations before a ready-to-use product is achieved which is cost-intensive and labor-intensive and introduces a higher degree of variation in DC characteristics. For this, efforts have been made to cryopreserve DC. Various reports using DC for cancer immunotherapy have demonstrated no differences regarding the morphology, phenotype, and function between cryopreserved and freshly generated DC [44–47]. However, to date, studies addressing the influence of cryopreservation on the characteristics of tolDC are limited.

Here, we report the development and optimization of a cryopreservation protocol which yielded a recovery of 78% and a viability of 75% of immature 1,25(OH)_2_D_3_-treated DC following a freeze-thaw cycle. Previously, other studies investigating the effects of cryopreservation on immunostimulatory DC demonstrated a recovery of 86% on average [44, 47, 48]. In this study, the recovery of tolDC appears to be lower as compared to immunostimulatory DC, albeit not statistically significant. However, since it has been demonstrated that 1,25(OH)_2_D_3_ can promote spontaneous apoptosis of mature DC* in vitro* [12], this can be attributed to a direct effect of 1,25(OH)_2_D_3_ and not of the cryopreservation procedure. Overall, our observations underscore the feasibility to cryopreserve tolDC without affecting the viability. Furthermore, no differences regarding the expression of activation markers, including costimulatory molecules, by 1,25(OH)_2_D_3_-treated DC could be observed following cryopreservation, indicative of a robust semimature phenotype of 1,25(OH)_2_D_3_-treated DC. In contrast, conventional DC display increased levels of CD86 expression and decreased levels of HLA-DR expression as compared to freshly generated mo-DC. Similarly, John et al. have shown that cryopreservation of immature mo-DC resulted in enhanced cell maturation but decreased endocytic activity and efficiency of adenoviral transduction [48]. Importantly, we have demonstrated that 1,25(OH)_2_D_3_-treated mo-DC are unable to activate allogeneic T cells as compared to conventional DC, irrespective of their maturation state or cryopreservation. Our study confirms previous findings by Raϊch-Regué and coworkers [14] demonstrating that 1,25(OH)_2_D_3_-treated DC of both healthy individuals and MS patients were able to induce T cell hyporesponsiveness following antigen-specific T cell stimulation. Indeed, following stimulation with 1,25(OH)_2_D_3_-treated DC myelin-reactive T cells were unable to respond to myelin-derived antigen rechallenge whereas their ability to respond to an unrelated antigen was maintained, underlining the potential of tolDC to induce hyporesponsiveness in an antigen-specific manner. As clinical translation is aimed for and the need for repetitive injections of tolDC for a prolonged clinical effect in EAE is reported [26], the use of cryopreserved tolDC would highly contribute to the large-scale production and the widely applicable use of tolDC. Recently, the same group reported the* in vivo* clinical efficacy of frozen tolDC in EAE as administration of frozen tolDC was able to abrogate EAE disease progression, mediated by an inhibition of antigen-specific reactivity, the induction of Treg and regulatory B cells (Breg), and the activation of immunoregulatory natural killer T (NKT) cells. Importantly, long-term treatment was well tolerated and exhibited a prolonged clinical beneficial effect [49].

Although the exact mechanism by which tolDC induce T cell hyporesponsiveness remains unclear, several mechanisms by which tolDC can induce tolerance and orchestrate T cell fate have been identified. First, it has been described that tolDC induce tolerance in a “DC-specific” manner through the induction of T cell anergy or apoptosis or deletion of autoreactive T cells. For this, the expression of so-called negative regulatory molecules has been identified to contribute to T cell tolerance. Indeed, Unger et al. observed an increased expression of these inhibitory molecules by 1,25(OH)_2_D_3_-treated DC contributing to the induction of T cell anergy [18]. However, in our hands, no pronounced expression of PD-L1 and ILT-3 by 1,25(OH)_2_D_3_-treated DC was observed as compared to conventional DC (Supplementary Figures  1A and B). Furthermore, we demonstrated that 1,25(OH)_2_D_3_-treated DC rendered PBL in a robust hyporesponsive state, even following rechallenge with fully mature conventional DC, thereby excluding tolDC-mediated induction of T cell anergy in agreement with previous reports [14]. Van Halteren and coworkers demonstrated that 1,25(OH)_2_D_3_-treated DC selectively can induce apoptosis in T cells stimulated via the HLA-peptide complex on the DC surface. Importantly, bystander T cells, either resting or activated by peptide-pulsed untreated DC, were unaffected [50]. In contrast, Raϊch-Regué et al. ruled out specific apoptosis of autoreactive T cells [14]. In addition, tolDC can also initiate immune tolerance via the induction or expansion of Treg (i.e., “infectious tolerance”). Indeed, several groups have demonstrated that 1,25(OH)_2_D_3_-treated DC are able to induce antigen-specific IL-10-secreting Tr1 cells, capable of suppressing proliferation of responder T cells* in vitro* [18, 51]. Furthermore, it was reported that the induction of Treg required repetitive boosting with 1,25(OH)_2_D_3_-treated DC and was mediated by PD-L1 [18] and/or membrane-bound TNF-*α* expressed on the tolDC surface [51]. However, we and others [14, 52] could not observe any differences in the frequency of Treg induced by 1,25(OH)_2_D_3_-treated DC. Altogether, further investigation is warranted in order to understand the complex cross talk between 1,25(OH)_2_D_3_-treated DC and T cells.

In conclusion, we deliver proof-of-principle that 1,25(OH)_2_D_3_-treated DC display a semimature phenotype and anti-inflammatory cytokine profile. Importantly, we demonstrate that 1,25(OH)_2_D_3_-treated DC induce antigen-specific T cell hyporesponsiveness to myelin-derived antigens. Furthermore, we report the feasibility of cryopreservation of 1,25(OH)_2_D_3_-treated DC. Since cryopreservation did not affect the viability, phenotype, and the allogeneic T cell-stimulatory capacity of 1,25(OH)_2_D_3_-treated DC, our results contribute to the large-scale production and the widely applicable use of 1,25(OH)_2_D_3_-treated DC. Recent efforts by the European COST (European Cooperation in Science and Technology) network A FACTT (action to focus and accelerate cell-based tolerance-inducing therapies) have resulted in initiatives to harmonize tolDC therapy in a cost-effective and efficient way [53]. We expect that the demonstrated feasibility of the cryopreservation of tolDC in this study is an important step forward in the field of tolDC vaccination and may lay the groundwork for the development of a new form of cellular immunotherapy for MS and other autoimmune diseases.

## Supplementary Material

Supplementary Figure 1: Phenotypic characteristics of *in vitro* differentiated iDC and cc-mDC from healthy controls and MS patients CD14+ monocytes were cultured for 6 days in the presence of IL-4 and GM-CSF or in the presence of IL-4, GM-CSF and 1,25(OH)_2_D_3_ to obtain conventional iDC (open dots) or 1,25(OH)_2_D_3_-treated iDC (filled triangles) respectively. On day 6, DC were stimulated with a cocktail of pro-inflammatory cytokines (i.e. cc-mDC) or left untreated (i.e. iDC). The mean fluorescence intensity (MFI) of (A) PD-L1, (B) ILT-3, (C) DC-SIGN, (D) CD14 and (E) CCR7 by DC of healthy controls (n=7) and MS patients (n=10) is determined by flow cytometry. Horizontal lines show the mean. Abbreviations used: MFI, mean fluorescence intensity; iDC, immature DC; cc-mDC, cytokine cocktail-matured DC.

## Figures and Tables

**Table 1:** Clinical details of the patients recruited into the study.

UPN	Gender	Age	MS-type	EDSS score	Disease duration (years)	Medication
MS-DC 001	F	51	RR-MS	3	14.5	Glatiramer acetate
MS-DC 002	M	52	SP-MS	5	19	None
MS-DC 003	F	27	CIS	0	1	None
MS-DC 004	M	35	RR-MS	3	2	Natalizumab
MS-DC 005	F	35	RR-MS	3	6	Natalizumab
MS-DC 006	M	33	RR-MS	3.5	14	Natalizumab
MS-DC 007	F	42	RR-MS	2	18	IFN-*β*
MS-DC 008	M	45	RR-MS	2	19	None
MS-DC 009	M	32	RR-MS	2.5	1	IFN-*β*
MS-DC 010	M	25	RR-MS	1.5	2	IFN-*β*

	M/F: 6/4	Median: 35 Range: 25–52	RR/CP: 8/1	Median: 3 Range: 0–5	Median: 10 Range: 1–19	

UPN, unique patient number; M, male; F, female; EDSS, expanded disability status scale; RR-MS, relapsing-remitting multiple sclerosis; SP-MS, secondary-progressive multiple sclerosis; and CIS, clinically isolated syndrome.

**Table:** (a) Healthy controls (*n* = 7) cc-mDC

Marker	Type	Fold change (cc-mDC/iDC)	MFI ± SD	*p* value^*∗*^	*p* value^*∗∗*^
CD86	Conventional DC	1.95	504 ± 99	*p* < *0.05*	*p* < *0.05*
	1,25(OH)_2_D_3_ DC	1.89	232 ± 96	*p* < *0.05*	

CD83	Conventional DC	4.44	89 ± 16	*p* < *0.05*	*p* < *0.05*
	1,25(OH)_2_D_3_ DC	1.64	36 ± 18	n.s.	

CD80	Conventional DC	3.21	61 ± 14	*p* < *0.05*	n.s.
	1,25(OH)_2_D_3_ DC	2.03	33 ± 11	n.s.	

HLA-DR	Conventional DC	1.36	153 ± 46	*p* < *0.05*	*p* < *0.05*
	1,25(OH)_2_D_3_ DC	1.43	86 ± 33	n.s.	

**Table:** (b) MS patients (*n* = 10) cc-mDC

Marker	Type	Fold change (cc-mDC/iDC)	MFI ± SD	*p* value^*∗*^	*p* value^*∗∗*^
CD86	Conventional DC	2.63	372 ± 90	*p* < *0.05*	*p* < *0.05*
	1,25(OH)_2_D_3_ DC	2.35	194 ± 77	*p* < *0.05*	

CD83	Conventional DC	2.30	59 ± 16	*p* < *0.05*	*p* < *0.05*
	1,25(OH)_2_D_3_ DC	1.17	31 ± 9	n.s.	

CD80	Conventional DC	2.42	41 ± 14	n.s.	n.s.
	1,25(OH)_2_D_3_ DC	1.76	24 ± 8	n.s.	

HLA-DR	Conventional DC	1.81	258 ± 61	*p* < *0.05*	*p* < *0.05*
	1,25(OH)_2_D_3_ DC	1.56	117 ± 59	n.s.	

CD14+ monocytes were cultured for 6 days in the presence of IL-4 and GM-CSF or in the presence of IL-4, GM-CSF, and 1,25(OH)_2_D_3_ to obtain conventional iDC or 1,25(OH)_2_D_3_-treated iDC, respectively. On day 6, DC were stimulated with a cocktail of proinflammatory cytokines (i.e., cc-matured DC (cc-mDC)) or left untreated (i.e., iDC). The mean fluorescent intensity (MFI) of costimulatory molecules, CD80 and CD86, of maturation marker, CD83, and of HLA-DR by various DC subsets of healthy controls (a) (*n* = 7) and MS patients (b) (*n* = 10) was evaluated. Results are expressed as fold change, calculated as the ratio between the MFI value of cc-mDC to the MFI value of iDC.

^*∗*^The *p* values indicated are calculated for cc-mDC versus iDC.

^*∗∗*^The *p* values indicated are calculated for conventional cc-mDC versus cytokine cocktail-matured 1,25(OH)_2_D_3_-treated DC.

MFI, mean fluorescence intensity; cc-mDC, cytokine cocktail-matured DC; iDC, immature DC; and n.s., nonsignificant.

**Table 3:** Immunophenotypic analysis of *in vitro* differentiated mo-DC from healthy controls before and after cryopreservation.

Healthy controls (*n* = 5) cc-mDC					
Marker	Type	Fold change (cc-mDC/iDC)	MFI ± SD	*p* value^*∗*^	*p* value^*∗∗*^
CD86	Conventional DC	1.03	637 ± 98	n.s.	*p* < *0.05*
	1,25(OH)_2_D_3_ DC	1.19	209 ± 155	n.s.	

CD83	Conventional DC	1.43	45 ± 10	*p* < *0.05*	*p* < *0.05*
	1,25(OH)_2_D_3_ DC	0.90	22 ± 6	n.s.	

CD80	Conventional DC	1.24	23 ± 7	n.s.	*p* < *0.05*
	1,25(OH)_2_D_3_ DC	1.00	15 ± 4	n.s.	

HLA-DR	Conventional DC	0.89	103 ± 21	n.s.	n.s.
	1,25(OH)_2_D_3_ DC	0.92	61 ± 36	n.s.	

CD14+ monocytes were cultured for 7 days in the presence of IL-4 and GM-CSF or in the presence of IL-4, GM-CSF, and 1,25(OH)_2_D_3_ to obtain immature conventional DC or 1,25(OH)_2_D_3_-treated DC, respectively. On day 7, iDC were frozen and stored at −80°C. Next, cryopreserved iDC were thawed, rested for 2 h at 37°C, and stimulated with a proinflammatory cytokine cocktail for 24 h (i.e., cc-mDC). The mean fluorescent intensity (MFI) of costimulatory molecules, CD80 and CD86, of maturation marker, CD83, and of HLA-DR by various DC subsets of healthy controls (*n* = 5) was determined Results are expressed as fold change, calculated as the ratio between the MFI value after maturation following a freeze-thaw cycle and the MFI value obtained at immature stage following a freeze-thaw cycle.

^*∗*^The *p* values indicated are calculated for cc-mDC DC after cryopreservation versus iDC after cryopreservation.

^*∗∗*^The *p* values indicated are calculated for conventional cc-mDC after cryopreservation versus 1,25(OH)_2_D_3_-treated cc-mDC after cryopreservation.

MFI, mean fluorescence intensity; cc-mDC, cytokine cocktail-matured DC; iDC, immature DC; and n.s., nonsignificant.

**Table 4:** Flow cytometric analysis of CD4+ CD25hi  FOXP3+ Treg and immunosuppressive cytokine-expressing Treg in cultures of PBL stimulated with MOG- and MBP-derived peptides in the presence or absence of conventional and 1,25(OH)_2_D_3_-treated DC.

	% CD4+ CD25hi FOXP3+ within CD3+ CD4+ T cells	% TGF-*β*+ within CD3+ CD4+ CD25− T cells	% IL-10+ within CD3+ CD4+ CD25− T cells	% IL-10+ TGF-*β*+ within CD3+ CD4+ CD25− T cells	*p* value^**∗**^
MOG/MBP peptide stimulated PBL + conventional iDC	2.29 ± 0.84	0.21 ± 0.10	0.15 ± 0.04	0.02 ± 0.01	n.s.

MOG/MBP peptide stimulated PBL + 1,25(OH)_2_D_3_ iDC	1.62 ± 0.66	0.17 ± 0.08	0.12 ± 0.02	0.02 ± 0.01	n.s.

^*∗*^The *p* values indicated are calculated for MOG- and MBP-derived peptides-stimulated PBL versus PBL cocultured with iDC in the presence of MOG- and MBP-derived peptides.

MOG, myelin oligodendrocyte glycoprotein; MBP, myelin basic protein; PBL, peripheral blood lymphocytes; iDC, immature DC; and n.s., nonsignificant.
