# Supplementary material for: Immunomodulatory Effects of 1,25-Dihydroxyvitamin D3 on Dendritic Cells Promote Induction of T Cell Hyporesponsiveness to Myelin-Derived Antigens
Source: J Immunol Res. 2016 Sep 14;2016:5392623. doi: 10.1155/2016/5392623 (PMC5039280; doi:10.1155/2016/5392623)
Supplement: Supplementary file 1 — Supplementary Figure 1: Phenotypic characteristics of in vitro differentiated iDC and cc-mDC from healthy controls and MS patients CD14+ monocytes were cultured for 6 days in the presence of IL-4 and GM-CSF or in the presence of IL-4, GM-CSF and 1,25(OH)2D3 to obtain conventional iDC (open dots) or 1,25(OH)2D3-treated iDC (filled triangles) respectively. On day 6, DC were stimulated with a cocktail of pro-inflammatory cytokines (i.e. cc-mDC) or left untreated (i.e. iDC). The mean fluorescence intensity (MFI) of (A) PD-L1, (B) ILT-3, (C) DC-SIGN, (D) CD14 and (E) CCR7 by DC of healthy controls (n=7) and MS patients (n=10) is determined by flow cytometry. Horizontal lines show the mean. Abbreviations used: MFI, mean fluorescence intensity; iDC, immature DC; cc-mDC, cytokine cocktail-matured DC. [file 5392623.f1.pdf]

Supplementary Materials

Supplementary Figure 1

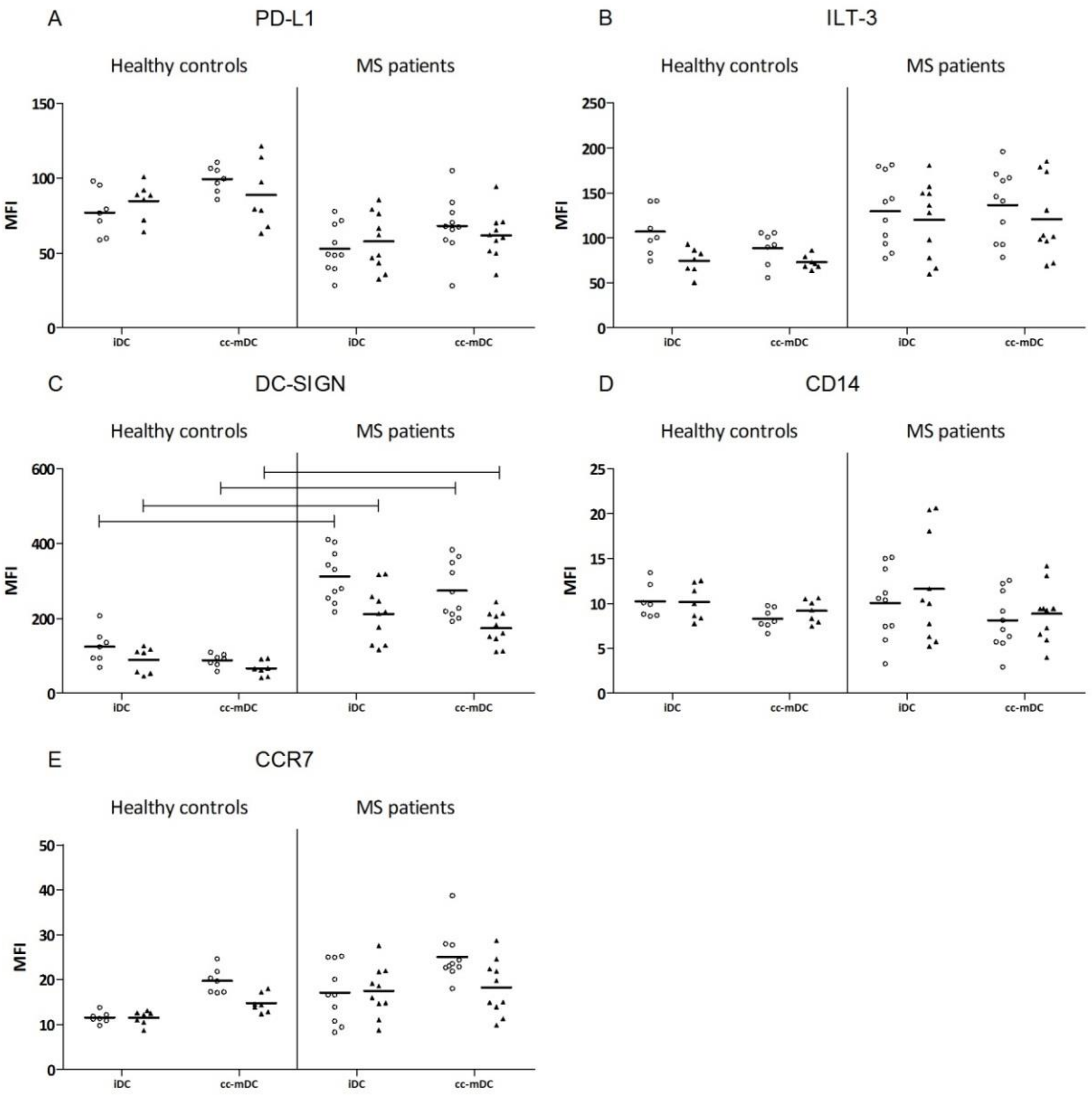

Supplementary Figure 1: Phenotypic characteristics of *in vitro* differentiated iDC and cc-mDC from healthy controls and MS patients

CD14+ monocytes were cultured for 6 days in the presence of IL-4 and GM-CSF or in the presence of IL-4, GM-CSF and 1,25$(OH)_2D_3$ to obtain conventional iDC (open dots) or 1,25$(OH)_2D_3$-treated iDC (filled triangles) respectively. On day 6, DC were stimulated with a cocktail of pro-inflammatory cytokines (i.e. cc-mDC) or left untreated (i.e. iDC). The mean fluorescence intensity (MFI) of (A) PD-L1, (B) ILT-3, (C) DC-SIGN, (D) CD14 and (E) CCR7 by DC of healthy controls (n=7) and MS patients (n=10) is determined by flow cytometry. Horizontal lines show the mean.

Abbreviations used: MFI, mean fluorescence intensity; iDC, immature DC; cc-mDC, cytokine cocktail-matured DC.
